# Locus-Specific Bisulfate NGS Sequencing of GSTP1, RNF219, and KIAA1539 Genes in the Total Pool of Cell-Free and Cell-Surface-Bound DNA in Prostate Cancer: A Novel Approach for Prostate Cancer Diagnostics

**DOI:** 10.3390/cancers15020431

**Published:** 2023-01-09

**Authors:** Olga Bryzgunova, Anna Bondar, Pavel Ruzankin, Anton Tarasenko, Marat Zaripov, Marsel Kabilov, Pavel Laktionov

**Affiliations:** 1 Institute of Chemical Biology and Fundamental Medicine, Siberian Branch Russian Academy of Sciences, 630090 Novosibirsk, Russia; 2 Sobolev Institute of Mathematics, Siberian Branch Russian Academy of Sciences, 630090 Novosibirsk, Russia; 3 Department of Mathematics and Mechanics, Novosibirsk State University, 630090 Novosibirsk, Russia

**Keywords:** cell-free DNA, cell-surface-bound DNA, methylation, locus-specific NGS sequencing, prostate cancer

## Abstract

**Simple Summary:**

Prostate cancer (PCa) is a global problem in the modern world. PCa has an almost 100% five-year relative survival rate at localized and regional stages and 31% at the appearance of distant metastases. Tumor development is always accompanied by a violation of DNA methylation, which could be detected even at the earliest stages of tumor development, not only in tumor tissue but also in cell-free DNA circulating in blood. The article describes a liquid biopsy approach for differential PCa diagnostics based on the data on locus-specific methylation of the two genes (GSTP1, RNF219) obtained with NGS of the total pool of blood cell-free DNA, including cell-free DNA from plasma and cell-surface-bound DNA, of PCa patients and healthy individuals. We offer a diagnostic approach that allowed a complete separation of cancer patients from healthy donors.

**Abstract:**

The locus-specific methylation of three genes (GSTP1, RNF219, and KIAA1539, also known as FAM214B) in the total pool of blood cell-free DNA, including cell-free DNA from plasma and cell-surface-bound DNA, of patients with prostate cancer and healthy donors was studied on the MiSeq platform. Our study found a higher methylation index of loci for total cell-free DNA compared with cell-free DNA. For total cell-free DNA, the methylation of GSTP1 in each of the 11 positions provided a complete separation of cancer patients from healthy donors, whereas for cell-free DNA, there were no positions in the three genes allowing for such separation. Among the prostate cancer patients, the minimum proportion of GSTP1 genes methylated in any of the 17 positions was 12.1% of the total circulated DNA fragments, and the minimum proportion of GSTP1 genes methylated in any of the 11 diagnostically specific positions was 8.4%. Total cell-free DNA was shown to be more convenient and informative as a source of methylated DNA molecules circulating in the blood than cell-free DNA.

## 1. Introduction

Prostate cancer (PCa) is the second-most commonly diagnosed cancer and the fifth leading cause of cancer death among men worldwide, with 1,414,259 new cancer cases and 375,304 deaths in 2020 [1]. PCa has an almost 100% five-year relative survival rate at localized and regional stages [2] and 31% at the appearance of distant metastases. Thus, detection of prostate cancer at the localized stages is necessary for successful treatment of PCa, to increase life expectancy, and to improve quality of life. To date, neither the prostate specific antigen (PSA) test [3] nor the digital rectal examination is 100% accurate, and because of overdiagnosis of prostate cancer (which can be as high as 80% [4]), since 2012, the U.S. Preventive Services Task Force (USPSTF) has no longer recommended PSA testing as a routine screening test for all men over the age of 50 [5]. The “Progensa PCA3” test, which determines the ratio of PSA mRNA to PCA3 lncRNA (long non-coding RNA) in the analysis of cell sediment from urine collected after rectal examination of the prostate [6], has also failed to meet expectations, although it has been shown to be more effective compared with analysis of PSA levels [7,8]. All this indicates the imperfection of the existing molecular methods for early diagnosis of prostate cancer and the relevance of the study described in this article.

Tumor development is always accompanied by a violation of DNA methylation. In fact, methylation at the fifth position of cytosines in CpG dinucleotides of tumor cells, including hypermethylation of promoter regions of tumor suppression genes or demethylation of regulatory regions of genes involved in oncogenesis, were found in all tested types of cancer, and could be detected even at the earliest stages of tumor development, not only in tumor tissue but also in cell-free DNA circulating in blood (cfDNA) [9,10,11,12]. In contrast to miRNA [13], DNA methylation directly regulates expression of the corresponding gene and thus is less susceptible to the unpredictable miRNA expression related to tissue specificity, or other events that are not associated with tumor development. This fact is especially important when cfDNA is used [14]. Liquid biopsy based on analysis of cfDNA methylation is currently approved by the FDA and used for colon cancer diagnostics (www.epiprocolon.com, www.epigenomics.com (accessed on 10.11.2022)).

Nevertheless, few cfDNA methylation-based real-time PCR liquid biopsy tests have been introduced to the medical diagnostics market since cfDNAs were first described in 1999 [15,16]. PCR tests have high diagnostic efficacy when their target sequence can be unmistakably defined and occurs only in the desired group. The difficulties in the design of such systems are precisely related to the search for such sequences. Along with low concentration of tumor-specific aberrantly methylated DNA (amDNA) in the pool of cfDNA and the technical steps (chemical conversion) that decrease the amount of the target, another phenomenon of heterogeneous methylation concerned with basic features of DNA methylation significantly complicates the selection of the specific methylated sequences for methylation-specific PCR [17]. This phenomenon has not yet been exhaustively studied [18], but it is known that each position of CpG dinucleotides in each DNA template molecule can be either methylated or unmethylated (see illustration at [19,20] and [21]). Allele specific methylation [19], and the variability/heterogeneity of tumor cells [22,23], which is related to stochastic epigenetic aberrations during oncotransformation of normal cells [24,25] or their phenotype transformation during the metastasis process [25], contribute to this phenomenon. In addition, variability between patients in the sets of epigenetic DNA aberrations in identical types of tumor increase intramolecular methylation diversity [25]. The molecular complexity of the loci of methylated tumor DNA and the presence of any set of methylated CpG in circulating DNA molecules [20,25] mean that study methods which do not yield information about each methylated cfDNA molecule (such as pyrosequencing) are of no use. To date, only bisulfite DNA sequencing using NGS technology provides information on the methylation profile of individual molecules. It must be mentioned that application of whole genome NGS bisulfite DNA sequencing for examining diagnostic amDNA sequences is limited by their low concentration and fragmentation [17] in cfDNA pool as well as insufficient coverage of CpG reach sequences. Indeed, loci with 10 CpG dinucleotides may potentially contain 2^10^ = 1024 methylated profiles, and whole genome sequencing with 200-fold coverage cannot yield satisfactory data on loci methylation in tissue, especially for the pool of circulating DNA. Loci-specific NGS bisulfite DNA sequencing provides possibilities to increase coverage of individual loci as required.

To increase the content of tumor-specific DNA in cfDNA samples, we propose the use not only of cfDNA from blood plasma, but also the total pool of cell-free DNA (tcfDNA) circulating in the blood, which includes cell-free DNA from plasma (cfDNA) and cell-surface-bound DNA (csbDNA) [26]. Previously, we demonstrated that the amounts of cell-surface-bound DNA (csbDNA) circulating in blood were at least comparable with the amount of cfDNA, and moreover, we observed that methylated DNA were readily found in the csbDNA fraction [27,28,29,30,31]. All of the above, including a simple method for isolation of the total pool of cell-free DNA circulating in the blood [32], have prompted us to execute the locus-specific NGS bisulfite DNA sequencing of three genes which have been shown to be methylated in PCa patients, in order to evaluate their intramolecular diversity and identify PCa markers, in addition to considering the design of diagnostic systems.

## 2. Materials and Methods

### 2.1. Study Population and Blood Collection

Blood samples of 18 healthy donors (HD) (control group: men with no evidence of prostate diseases, PSA level <2.8 ng/mL) were recruited in ICBFM SB RAS clinics, and 19 patients with prostate cancer were recruited at the Novosibirsk Regional Oncology Dispensary (Novosibirsk, Russia) (Table 1). In the patients with prostate cancer, tumor staging was performed according to the TNM classification and the analysis of prostate biopsy. All the patients with prostate cancer were recruited to the study before undergoing any treatment and all had tumors localized in the prostate, without lymph nodes or distant metastases (T2-3NxMx). The study was carried out in compliance with all principles of voluntariness and confidentiality in accordance with the “Fundamentals of Healthcare Legislation” of the Russian Federation. The study was approved by the ethics committees of the ICBFM SB RAS and the Novosibirsk Regional Oncology Center (N 15309-01 from 22 December 2008) and written informed consent was provided by all participants.

Within 4 h of peripheral blood being taken and collected in Vacutainer tubes containing K3EDTA (BD, cat. No. 368589), the blood was processed for isolation of cfDNA and tcfDNA [21,32]. To obtain tcfDNA, DNA Detachment Buffer (DDB) disrupting interaction of DNA with cell surface [32] was added to the blood and after pelleting of the blood cells, supernatant containing tcfDNA was obtained. Both cfDNA and tcfDNA were isolated using column-based protocol as described below.

### 2.2. DNA Extraction, Quantification, and Bisulfate Conversion

Genomic DNA from human male leukocytes was used to prepare standards for calibration curves using TaqMan real-time PCR and unmethylated DNA controls (completely unmethylated DNA). The cfDNA and tcfDNA from 1 mL and 2 mL of blood, respectively, was isolated using a Blood Plasma DNA Isolation Kit (BioSilica Ltd., Novosibirsk, Russia) in accordance with the manufacturer’s instructions and then eluted into 240 µL nuclease-free water. After the extraction, 225 µL of the DNA sample was mixed with 5 µL of glycogen (20 mg/mL, Fermentas, Vilnius, Lithuania) and 1/10 volume of 50 mM triethylamine, and then precipitated with 5 volumes of acetone [33]. After the precipitation, the cfDNA and tcfDNA were reconstituted in 52 µL of water. The DNA aliquot from this step (2 µL) was diluted 6 times and quantified using LINE1 TaqMan PCR (see below). The DNA samples were frozen and stored at −20 °C until use.

Bisulfite treatment was performed using EZ DNA Methylation-Gold™ Kit (ZymoResearch, Irvine, CA, USA) in accordance with the manufacturer’s instructions. All samples (50 μL) of cfDNA and tcfDNA (~10–30 ng) or similar amounts of genomic DNA were treated simultaneously. The bisulfite-treated blood cfDNA and tcfDNA was eluted from DNA spin columns in 32 μL of elution buffer. DNA aliquot (2 µL) was used to quantify DNA using LINE1 TaqMan PCR, and 30 µL were stored at −20 °C until loci-specific PCR amplification.

The TaqMan real-time PCR targeting the LINE1 elements was designed to amplify both innate and bisulfite-treated DNA in order to control DNA loss and input in loci-specific PCR. Genomic DNA from human male leukocytes before and after bisulfite conversion was used to receive calibration curves for different DNA samples. The total volume of the PCR was 30 μL using 5 μL DNA and 15 μL 2× QuantiTect Kit. Reaction conditions are indicated in Table 2. Real-time PCR was performed on an iCycler iQ5 Real-Time PCR Detection System (Bio-Rad, Hercules, CA, USA).

### 2.3. Amplification of Selected Loci

During loci-specific PCR, PCR products were barcoded with a pair of 8 b.p. unique indexes encoding the patient. Barcode sequences had been previously published [34].

The loci of interest were amplified using Hotstar Taq polymerase (Qiagen, Hilden, Germany), according to the manufacturer’s instructions, with minor changes. Briefly, 5 μL of bisulfite-treated total blood cell-free DNA was amplified under the conditions indicated in Table 2. The unique barcodes are listed in Table 3. Reactions were performed using Veriti Thermal Cycler (Applied Biosystems, Waltham, MA, USA). After the amplification, the PCR products were stored at −20 °C until use.

For each PCR, positive (5 ng genomic DNA from human male leukocytes after bisulfite conversion) and negative (no-template DNA) controls were used. The PCR products were quantified using the DNA 500 kit on the Shimadzu MCE-202 MultiNA (Shimadzu Corporation, Kyoto, Japan).

### 2.4. Preparation of Sequencing Libraries

To prepare DNA libraries, PCR products of one gene and one patient group were pooled in equimolar amounts calculated using data from the Shimadzu MCE-202 PCR product assay. Then, the PCR products were purified in 2% agarose gel using a GenJet Kit (Thermo Scientific, Waltham, MA, USA) and quantified using a Qubit fluorometer (Life Technologies, Carlsbad, CA, USA) for library preparation.

DNA libraries were prepared using a NEBNext Ultra DNA Library Prep Kit (New England BioLabs, Ipswich, MA, USA) in accordance with the manufacturer’s protocol. The quality of the libraries was evaluated using a High Sensitivity DNA Agilent chip run on the Agilent 2100 Bioanalyzer (Agilent Technologies, Santa Clara, CA, USA) to confirm the size and concentration of PCR products. The libraries were analyzed using a 150 + 150−nt paired-end Illumina MiSeq sequencing run (in SB RAS Genomics Core Facility (ICBFM SB RAS, Novosibirsk, Russia). PCR products that were produced on completely methylated and completely unmethylated DNA were used as sequencing controls.

### 2.5. NGS Data and Statistical Analysis

The CpG methylation status of the DNA loci was analyzed using BiQ Analyzer HT Software with a minimal conversion rate of 0.95 and disallowing readings with unrecognized sites.

To assess the representation in groups of patients and healthy donors of sequences with methylated or unmethylated cytosine, we introduced the variables that correspond to the proportions of such sequences. “C” represents methylated cytosine and “T” represents unmethylated cytosine (uracil/thymidine after chemical conversion/amplification). The variables GSTP1.C1–GSTP1.C17 correspond to the proportions in the total number of sequences with methylated cytosine in the investigated locus of the GSTP1 gene at positions from 1 to 17, respectively. Similar variables were introduced for the studied loci of the RNF219 and KIAA1539 genes: RNF219.C1–RNF219.C17 and KIAA1539.C1–KIAA1539.C5.

To assess the sequences with methylated or unmethylated cytosine in two different positions of the same molecule, we introduced variables corresponding to the proportions of such sequences. For example, the variable RNF219.C4.T10 represents the proportion of sequences with methylated cytosine in the fourth position and unmethylated cytosine in the tenth position of the studied RNF219 gene locus. Methylation at one of the positions and the paired (“linked”) methylation positions of the GSTP1, RNF219, and KIAA1539 gene loci was described by 1167 = 17 + 17 + 5 + (17 × 16/2 + 17 × 16/2 + 5 × 4/2) × 4 variables. Here, 17 + 17 + 5 is the number of variables describing methylation of a single position; 17 × 16/2, 17 × 16/2, and 5 × 4/2 are the numbers of choices of two positions from 17, 17, and 5 positions in the GSTP1, RNF219, and KIAA1539 genes, respectively; 4 is the number of variables for each pair of chosen positions.

A statistical analysis of the distribution of variables in the comparison groups of healthy donors and prostate cancer patients was performed. The distributions of the variables corresponding to one methylation position for the HD and PCa groups were plotted on boxplots. The boxes depict the medians and the 1st and 3rd quartiles.

For each of the introduced variables corresponding to the methylation, the comparison groups were compared using the exact Mann–Whitney test. For the sake of convenience, we report the adjusted *p*-values, which are the p-values multiplied by the number of comparisons (1167) according to Bonferroni’s approach. The differences corresponding to *p*-values < 0.05/1167 (i.e., where the adjusted *p*-values were < 0.05) were considered significant.

As well as the *p*-values, we calculated the following prediction accuracy measures for the comparisons: sensitivity for 100% specificity, specificity for 100% sensitivity, accuracy for cross-validation, sensitivity for cross-validation, specificity for cross-validation, and ROC AUC with 95% DeLong’s confidence interval for cross-validation. DeLong’s confidence intervals are not reported when ROC AUC = 1, since they are always (1, 1) in that case. We used leave-one-out cross-validation and the predicted values were computed using logistic regression with weights balancing the comparison groups. The cross-validation accuracy, sensitivity, and specificity were calculated for the threshold 0.5. If a complete separation (100% sensitivity for 100% specificity for some cutoff value) was attained for a variable, then the accuracy, sensitivity, specificity, and ROC AUC are reported as 100%.

Additionally, the cutoff values are reported for the variables. The cutoff value is an estimate of the threshold which may be appropriate for separation of the comparison groups. In the case of complete separation, the cutoff value is the geometric mean of the two closest values, one being taken from one comparison group and the other from the other comparison group. If one of the two closest values is zero, then the arithmetic mean is reported instead. The reported ratio is the ratio of the two closest values if both of them are nonzero. If complete separation is not attained, then the cutoff is reported as the threshold maximizing sensitivity + specificity, and the ratio is not reported. Finally, the mean values in the comparison groups are reported.

The analysis was performed using R v. 4.1.1 (R Core Team, Vienna, Austria).

## 3. Results

As we mentioned in the introduction, tcfDNA could represent a valuable source of tumor-specific aberrantly methylated circulating DNA. This pool of DNA includes circulating cfDNA, which, as we have shown earlier, contains tumor-specific aberrantly methylated DNA [21]. To evaluate the usefulness of tcfDNA we will compare current data with those previously published [21].

The flowchart of the study is shown in graphical abstract and Supplementary Figure S1.

After isolation, bisulfate conversion, and quantification of cfDNA and tcfDNA, it was used for locus-specific amplification, preparation of DNA libraries for sequencing, NGS sequencing, and data processing.

Because the tcfDNA processing protocol before loci-specific sequencing involves many stages, it was necessary to assess the loss of DNA at each step. Thus, to control DNA input in target bisulfite sequencing, methyl-independent TaqMan PCR of LINE1 elements was used. The accuracy (variation coefficient) of real-time PCR was 12%, sensitivity was 5 pg of genomic DNA, and the efficacy was 97.4–99.5%. Each sample used in the study contained at least 3.3 ng/mL DNA in the initial blood, with the typical yield of total cell-free DNA after bisulfite conversion varying within a range of 70–80%. After locus-specific amplification with methyl-independent primers, at least 50 ng DNA was obtained for each locus.

After equimolar mixing of PCR products and purification using gel-electrophoresis, a minimum of 100 ng of the total mixture of PCR products was used for library preparation based on Agilent 2100 Bioanalyzer data. During loci-specific PCR, the PCR products were barcoded with a pair of 8 b.p. unique indexes encoding the patient as recommended by Caporaso [34], to avoid errors in sequencing and data analysis.

In order to exclude the appearance of chimeric DNA between the molecules of PCa patients and healthy donors, PCR products were coded using eight 5′ forward (Xn) and twelve reverse primers (Yn), so that groups of samples from prostate cancer patients and healthy donors did not contain common barcodes. Sequences obtained using NGS that had primer combinations which were not present in the preparation of the libraries were considered chimeric and excluded from the data analysis.

A total of 6.3, 9.0, and 9.2 million reads were obtained for GSTP1, RNF219, and KIAA1539, respectively, using paired-end 250 bp sequencing. The quality of all libraries was high, and the percentage of uniquely-mapped reads varied within the range of 80–85%. Selected loci were sequenced with coverage ranging from 23,509 to 143,953.

After primary data analysis, the number of molecules with similar methylation profiles for every gene in each patient was calculated.

**Figure 1:**
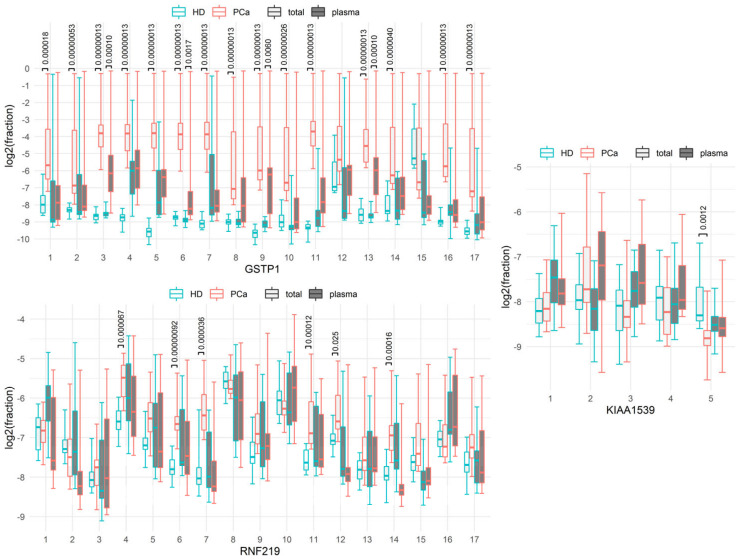
Individual CpG dinucleotides methylation level at different positions of the studied loci. Methylation level of cfDNA in accordance to [21] presented as grey boxplots. HD and PCa groups are marked in blue and red, correspondingly. The boxes depict medians with the 1st and 3rd quartiles. The whiskers depict minimums and maximums. The adjusted p-values are reported for the differences (i.e., the *p*-values multiplied by 1167), where the adjusted *p*-values are <0.05.

The proportions of sequences with methylated cytosine at each of the positions of the molecule in the HD and PCa groups are presented in boxplots (Figure 1).

Comparison of the current data with those previously obtained [21] demonstrates a much higher level of methylation of some loci in the tcfDNA pool compared with cfDNA. In fact, when cfDNA was studied, elevation of only C9 methylation of GSTP1 gene locus was characteristic for PCa patients [21]. In that study, the methylation was compared between the PCa group and the combined HD and benign prostatic hyperplasia group. When comparing the PCa and HD groups from the data of that study, the significant differences in the levels of individual cytosines were observed only for C3, C6, C9, and C13 of the GSTP1 gene (see Figure 1 and Table 4). In the pool of tcfDNA, a number of cytosine methylations (for example, C3–C9, C11, et al.) drastically differed between PCa patients and healthy donors. The total methylation levels of individual cytosines of the RNF219 and KIAA1539 genes in both blood DNA fractions in the studied groups of patients did not differ significantly, with the exceptions of C4, C6, C7, C11, C12, and C14 in tcfDNA of the RNF219 gene and C5 in tcfDNA of the KIAA1539 gene. Table 4 shows that tcfDNA pool data provided greater sensitivities and specificities for predicting PCa than cfDNA pool data did.

**Figure 2:**
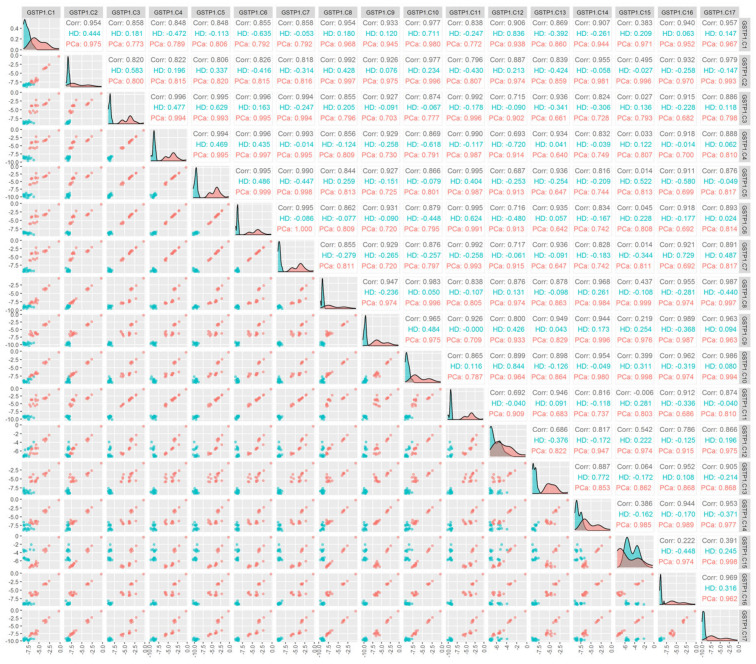
Correlation between the methylation of the individual cytosines of the studied locus of the GSTP1 gene in the pool of tcfDNA. Binary logarithms of the variables were used for all the plots. The diagonal cells present the density plots for the PCa (red) and HD (blue) groups for each of the variables. Each diagonal cell has its own y-axis scale, which is not labeled. “HD” and “PCa” denote the respective Pearson correlations for each of the groups. “Cor” denotes the Pearson correlation for all the groups combined; the correlations are calculated for the binary logarithms of the variables. The cells below the diagonal present scatterplots for each pair of variables.

An analysis of the correlation between the methylation status of individual cytosines for the GSTP1 gene in tcfDNA (Figure 2, Pearson correlation coefficients for logarithms of concentrations were computed) demonstrates that the methylation of individual cytosines correlates in PCa patients (namely, C1–C2,8–10,12,14–17; C2–C8–10,12–17; C3–C4–7,11,12; C4–C5–7,11,12; C5–C6,7,11,12; C6–C7,11,12; C7–C11,12; C8–C9,10,12,14–17; C9–C10,12,14–17; C10–C11,14–17; C11–C12; C12–C14–17; C14–C15–17; C15–C16,17; r > 0.9). Moreover, in PCa patients for C6–C5 and C8–C15, the correlation coefficient constituted r = 0.999, and for the C7–C6 combination, r = 1.000, whereas for HD, a high correlation (r > 0.8) was present only for C12 methylation with C1 and C10.

The comparisons of cytosine methylation or simultaneous methylation of two cytosines between PCa patients and the group of HD in tcfDNA are presented in Table 5.

Note that sequences methylated at diagnostically significant positions are well represented, and the cutoff level varies in the range 0.17–1%.

**Figure 3:**
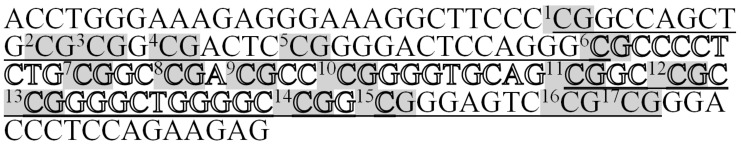
Sequence of the promoter region of the GSTP1 gene. The studied CpG dinucleotides are highlighted in gray and numbered.

Based on the data obtained, we tried to calculate the possibility of creating TaqMan PCR primers and probe. The analysis included only combinations of sequences that allowed separation of the studied groups of donors with 100% sensitivity at 100% specificity. The best results were obtained for the GSTP1 gene. Conventionally, the studied region of the GSTP1 gene was divided into three parts (Figure 3) because of the need to use two primers when creating a system for TaqMan PCR (the regions are underlined: Forward C1–C6, Reverse C11–C17) and a probe (the region is written with a contour C6–C15). The regions partially overlap.

First, the best combinations of each of the parts were selected separately (Table 6). The combinations of cytosines used for further selection of the PCR probe and primers are highlighted in bold (Table 7).

As can be seen from Table 7, the numbers of tcfDNA molecules with the required combination of cytosines slightly exceed 6% on average, which is insufficient for detection by TaqMan PCR in terms of the amount of DNA in the blood. Theoretically, if the introduction of “the errors” (bold and underlined) into the center of primers and probe did not decrease PCR efficiency, the number of detected molecules could theoretically increase (Table 8).

However, the molecules with the cytosine combinations presented in Table 8 were not found in the tcfDNA pool at all.

Unfortunately, for the RNA219 and KIAA1539 genes, no combinations of molecules in the tcfDNA pool were found that are suitable for PCR primers, and that exceeded 0.5% in number (data not shown).

## 4. Discussion

It is known that various epigenetic changes underlie the development of prostate cancer [35] and a promising approach in the search for biomarkers of prostate tumors is the study of DNA methylation, including the analysis of cell-free DNA circulating in blood (liquid biopsy) [36]. To date, there are no diagnostic tests for prostate cancer that improve on the routine screening for PSA [37], despite the emergence of the PCA3 test and some others [38,39].

Initially it was assumed that calculation of the methylation index (the average ratio of methylated and unmethylated CpG) would be useful in PCa diagnostics. Although such an analysis showed low specificity, both when using tissue samples and various biological fluids, it did help to determine a set of possible potential aberrantly methylated genes – for example, GSTP1, SFRP2, et al. [40,41]. To date, the deepest method of genetic analysis of aberrant changes is undoubtedly NGS, which can provide accurate and complete information on the methylation status of individual DNA molecules.

In this work, to identify aberrantly methylated DNA molecules that appear in the blood during the development of prostate cancer, the methylation status of individual cytosines in the CpG dinucleotides of the promoter regions of the GSTP1, RNF219, and KIAA1539 genes was analyzed using locus-specific NGS sequencing. The methylation status of these three genes has been found to change with the development of prostate tumors [29,42,43]. Many researchers pinned great hopes on the development of diagnostic testing based on the analysis of the methylation status of cytosines in the promoter region of the GSTP1 gene, since it was assumed that in genomic DNA obtained from prostate tumor tissue, cytosines in CpG dinucleotides are methylated in almost 100% of cases. However, using bisulfite sequencing, it was shown that this gene is aberrantly methylated in 86% of tumor tissues of the prostate [44]. Moreover, allele-specific methylation [19] and nontotal DNA methylation found in many studies [20,21] decreases the attractiveness of using aberrantly methylated DNA as tumor markers.

It is also worth mentioning the concentration of tumor-specific DNA in the pool of cfDNA from blood. It is known that 1 mL of blood plasma from a cancer patient contains about 1500 diploid genome equivalents (GE), which is about 10 ng of DNA [45]. Few studies have presented data regarding higher concentrations of cfDNA in cancer patients, and others have failed to confirm this [46]. Starting from the previously mentioned amount of cfDNA and a standard blood sample equal to 10 mL, results of about 6000 GE (12 × 10^3^ molecules per region or gene) can be obtained in 4 mL of plasma for diagnostic purposes (including duplicates). The concentration of tumor-specific DNA in blood plasma varies from 1% to 10%, thus 60–600 GE are available for the assay. Considering the nonuniform presentation of genomic DNA in circulation [46,47] and the aforementioned complications with methylated DNA, as well as specificity and sensitivity PCR, the detection of such markers represents a complicated task.

One approach to overcome these complications is an increase in the amount of cfDNA. Previously we have found the presence of cfDNA bound with the surface of blood cells in amounts at least comparable with that of cfDNA from plasma [48], and aberrantly methylated DNA, suitable for sequencing, were found in this fraction [27,29,49]. As we have shown the possibility of using of NGS locus-specific sequencing of cfDNA to reveal tumor-specific methylation [21], we wondered whether tcfDNA would also be applicable for identifying tumor-specific aberrantly methylated DNA molecules using locus-specific NGS.

It should be mentioned that starting from the theoretical considerations regarding the aforementioned cfDNA and tcfDNA features and taking into account the proportion of tumor-specific DNA in the total pool of tcfDNA circulating in the blood [50], we can say that even 10^4^ readings per studied locus per sample provide at least 100-fold coverage of tcfDNA and reliable detection of a single tumor-specific molecule, whereas total bisulfate NGS sequencing is unable to provide the required coverage.

The data obtained demonstrate a higher proportion of methylated DNA in the pool of tcfDNA (Figure 1) and significantly higher diagnostic potential of tcfDNA. This statement is true for the GSTP1 and RNF219 genes.

Overall, the GSTP1 gene loci studied represent the best potential marker: the methylation status of a few individual cytosines differentiated PCa patients from HD with 100% sensitivity and 100% specificity. At the same time, positions with such high potential diagnostic value were not found in the studied loci of other genes. Furthermore, within the studied loci, we also considered paired methylation of cytosines – namely, simultaneous methylation, methylation of one and the absence of methylation of the second, or the absence of methylation of two specific cytosines in one DNA molecule [21]. This approach improves the accuracy of a possible diagnostic system. It should be noted that most of the cytosines in the loci of the studied tcfDNA genes in the blood of patients with prostate cancer are methylated, whereas in healthy donors they are not.

When analyzing the data regarding the frequency of representation of molecules with a marker methylation profile in circulation and the cutoff level (Table 5), the representation of molecules with such sequences is generally close to the percentage of tumor-specific DNA in circulation [45].

Today, there are already diagnostic methods using standard approaches that allow reliable identification of the differences between cancer patients and healthy people. For example, NGS with coverage at one locus of 10,000 or more is able to detect the difference between disease and health in addition to PCR with a single nucleotide extension of the primer followed by the detection of PCR products using mass spectrometry (for example, Agena Bioscience starting from 10–15 ng DNA can detect 1% variant allele frequency [https://agenabio.com/products/panels/cancer-solutions/] (accessed on 15.10.2022)).

Methylation assay of the GSTP1 gene appears to be the most suitable approach for detecting PCa. Indeed, the presence of 147 variables (11 of which correspond to single and 136 simultaneously to two methylated cytosines in the same molecule) for the GSTP1 gene and 110 variables (corresponding to two simultaneous methylated cytosines in one molecule) for the RNF219 gene, that exclusively distinguish patients with prostate cancer, with an adjusted *p* = 0.00000013, suggests that increasing the sample size will not change the situation, and at least some of the variables will retain their diagnostic efficiency in the analysis of single molecules or in the analysis of a set of diagnostic molecules. For the KIAA1539 gene, the best indicators of sensitivity and specificity for dividing the study groups of patients were achieved only for two variations and amounted to 89.5% and 61.1%, respectively (Supplementary Table S1).

Thus, the use of a large data set, which allows cancer patients to be distinguished from healthy people with 100% sensitivity and specificity, provides great opportunities for choosing the best analytical systems. Today, there are several platforms capable of detecting aberrant methylated DNA, such as Illumina HumanMethylation27 Bead-Chip, Illumina HumanMethylation 450 BeadChip array, DREAMing, etc. [51,52,53,54,55,56,57], but only NGS provides accurate information on the methylation status of separate cytosines in one DNA molecule.

In a modern diagnostic laboratory, to detect the simultaneous methylation of two cytosines in one DNA locus, a new-generation NGS can be used in combination with target enrichment protocols [58]; in addition, platforms based on mass spectrometry, such as MassARRAY from Agena Bioscience Inc., are also suitable.

The development of diagnostic systems based on the analysis of aberrant DNA methylation has several advantages over, for example, miRNA: (1) the process of change in the DNA methylation is almost always observed in tumor tissues [59,60]; (2) because methylation change regulates only one specific gene and is not involved in the regulation of the whole gene network, we can regard it as a more “accurate” tumor marker [61,62]; (3) because of the heterogeneous origin of the tumor, the advantage of DNA lies in its amount proportional to the number of cells, whereas, for example, for RNA, a small subpopulation of cells with high transcription rates can mask the profiles of others; and (4) the use of methylated extracellular DNA as a source of tumor markers is also advisable in connection with its increased stability relative to unmethylated DNA, which leads to a longer circulation time in the bloodstream [63], and theoretically should lead to an increase in the amount of a detectable tumor marker in the blood with the growth of tumors, and thus improve the accuracy of diagnostic systems.

It should be noted that the number of profiles obtained in this study exceeds the level of the estimated data obtained from the tcfDNA concentration, which may be related to the sequencing technology used. Of course, the data obtained require verification, which can be performed both by increasing the number of patients and by use of reference methods. However, the large set of data obtained suggest that at least some of the detected markers will have the declared characteristics, which may increase the accuracy of prostate cancer diagnosis.

It should also be noted that the characteristic features of the methylation marker sequences (simultaneous methylation of cytosines of the studied loci) that we discovered may not only reflect the features of DNA methylation in patients with prostate cancer (Figure 2), but also the features of gene expression regulation, DNA packaging, and the work performed by tumor methylases.

Unfortunately, despite all the advantages, there is one disadvantage that prevents the creation of relatively inexpensive TaqMan PCR kits for PCa diagnostics in clinics. This relates to the small number of molecules with the specific methylation profile that anneal with primers and probe. However, methylation-independent amplification of GSTP1 loci combined with subsequent single nucleotide elongation from multiple primers, mass spectrometry, and evaluation of cut off level, may have potential.

The more abundant presence of methylated DNA in the pool of tcfDNA [27,64] can also lead to an increase in the accuracy of the diagnostic test. This fact is supported by the data that long DNA fragments (more than 10 kb) are mainly associated with the surface of blood cells, as well as the overrepresentation of methylated DNA in cell-surface-bound DNA [65].

## 5. Conclusions

In conclusion, it should be noted that use of the locus-specific NGS method not only provides data on the methylation of individual molecules of the RNF219, KIAA1539, and GSTP1 genes in the tcfDNA of patients with prostate cancer and healthy donors, but also enables differentiation of both studied groups of patients with 100% sensitivity and specificity.

The reason for the more abundant presence of methylated DNA in the pool of tcfDNA is not clear, but is obviously related to the binding of methylated DNA with cells. The reasons for such binding could be related to fragmentation, methylation level, methyl-binding proteins, or the interaction of histones with blood cell surface receptors.

The interconnection of methylation profiles with regulation of gene expression and its impact in tumor development remain to be studied.

## Figures and Tables

**Table 1:** Overview of the study population.

Characteristic	Groups	
	Prostate Cancer Patients *n* = 19	Healthy Donors *n* = 18
**Age**		
Mean ± SD	67.7 ± 7.0	61.6 ± 6.6
Range	55–77	53–74
**Total PSA, ng/mL**		
Mean ± SD	17.3 ± 12.9	1.2 ± 0.7
Range	4.8–48.7	0.2–2.3
**Tumor stage**		
T2bNxMx	6	N/A
T2bNxMx	5	
T3aNxMx	4	
T3bNxMx	3	
**Gleason scale**		
Unknown	1	N/A
4–5	1	
5	2	
5–6	3	
6	6	
7	4	
8	2	

**Table 2:** PCR conditions and primer’s sequences.

Target’s Name	Primer’s Sequence (without Barcodes)	Primers/Probe Concentration, nM	Length of PCR Product, b.p.	Length of PCR Product without Barcodes, b.p.	CG Number	1× Buffer Composition	PCR Conditions
LINE1-For LINE1-Rev LINE1-Probe	5′-AATGGAAGATGAAATGAATGAAATGA-3′	600/300	-	155	-	BioMaster qPCR Mix from Biolabmix (Novosibirsk, Russia)	95 °C-3 min, (95 °C-15 s, 60 °C-60 s) ×40
	5′-TTCCATTCTCCCCATCACTTTCA-3′						
	5′-FAM-GAGAAGGGAAGTTTAGAGAAAAAAGAAT-FQ-3′						
RNF219-For RNF219-Rev	5′-(Y1-12)GTGATTGTGGGTATAGTTATAAAA-3′	600	177	161	17	Hotstart PCR buffer with additional MgCl_2_ (final concentration 5 mM), 1 mM dNTPs and 0.65 units of Hotstart Taq polymerase	95 °C-15 min (95 °C-60 s, 58 °C-45 s, 72 °C-60 s) ×50
	5′-(X1-8)ACTACCCCCATCTCCCAAAA-3′						
KIAA1539-For KIAA1539-Rev	5′-(X1-8)AGGAAGGAGGAGATAAAGTGAT-3′	600	105	89	5		
	5′-(Y1-12)CCCCTCTAAACTTATCATCACA-3′						
GSTP1-For GSTP1-Rev	5′-(Y1-12)ATTTGGGAAAGAGGGAAAGGTT-3′	600	158	142	17		
	5′-(X1-8)CTCTTCTAAAAAATCC-3′						

**Table 3:** Barcodes information.

BARCODES	With Forward or Reverse Primer the Exact Barcode Was Used for Each Target		
	RNF219	KIAA1539	GSTP1
X1: TAGATCGC, X2: CTCTCTAT, X3: TATCCTCT, X4: AGAGTAGA, X5: ACTGCATA, X6: AAGGAGTA, X7: CTAAGCCT, X8: CCTCTCTG	Reverse	Forward	Reverse
Y1: TCGCCTTA, Y2: CTAGTACG, Y3: TTCTGCCT, Y4: GCTCAGGA, Y5: AGGAGTCC, Y6: CATGCCTA, Y7: GTAGAGAG, Y8: CCTCTCTG, Y9: AGCGTAGC, Y10: CAGCCTCG, Y11: TGCCTCTT, Y12: TCCTCTAC	Forward	Reverse	Forward

**Table 4:** Comparison of accuracies corresponding to the tcfDNA pool and cfDNA one for GSTP1 gene.

	*p*-Value × 1167	Means, % (PCa/HD)	Sensitivity for 100% Specificity, %	Specificity for 100% Sensitivity, %	CV Accuracy, %	CV Sensitivity, %	CV Specificity, %
GSTP1.C1, total	0.000018	8.36/0.483	68.4	83.3	86.5	84.2	88.9
GSTP1.C1, plasma	>1	5.83/5.73	5.6	12.5	0	0	0
GSTP1.C2, total	0.00000053	7.72/0.321	94.7	88.9	94.6	94.7	94.4
GSTP1.C2, plasma	>1	5.42/5.11	5.6	6.2	11.8	0	25.0
GSTP1.C3, total	0.00000013	12.0/0.247	100	100	100	100	100
GSTP1.C3, plasma	0.00010	7.74/0.293	88.9	68.8	85.3	83.3	87.5
GSTP1.C4, total	0.00000013	11.8/0.235	100	100	100	100	100
GSTP1.C4, plasma	>1	9.67/4.16	11.1	18.8	50.0	22.2	81.2
GSTP1.C5, total	0.00000013	12.1/0.133	100	100	100	100	100
GSTP1.C5, plasma	>1	8.46/1.41	16.7	37.5	55.9	22.2	93.8
GSTP1.C6, total	0.00000013	11.9/0.234	100	100	100	100	100
GSTP1.C6, plasma	0.0017	5.36/0.213	55.6	56.2	82.4	83.3	81.2
GSTP1.C7, total	0.00000013	12.0/0.187	100	100	100	100	100
GSTP1.C7, plasma	> 1	5.48/5.89	5.6	6.2	50.0	94.4	0
GSTP1.C8, total	0.00000013	6.95/0.196	100	100	100	100	100
GSTP1.C8, plasma	>1	5.77/0.198	61.1	12.5	76.5	61.1	93.8
GSTP1.C9, total	0.00000013	8.62/0.124	100	100	100	100	100
GSTP1.C9, plasma	0.0060	6.04/0.179	77.8	25.0	85.3	77.8	93.8
GSTP1.C10, total	0.00000026	8.23/0.217	94.7	94.4	94.6	94.7	94.4
GSTP1.C10, plasma	>1	5.20/0.222	22.2	6.2	64.7	38.9	93.8
GSTP1.C11, total	0.00000013	12.6/0.155	100	100	100	100	100
GSTP1.C11, plasma	>1	5.73/0.497	11.1	18.8	55.9	27.8	87.5
GSTP1.C12, total	0.63	9.10/1.61	36.8	61.1	64.9	42.1	88.9
GSTP1.C12, plasma	>1	9.30/5.07	5.6	43.8	47.1	5.6	93.8
GSTP1.C13, total	0.00000013	8.42/0.274	100	100	100	100	100
GSTP1.C13, plasma	0.00010	11.0/0.258	77.8	81.2	85.3	77.8	93.8
GSTP1.C14, total	0.0000040	8.50/0.420	68.4	94.4	91.9	89.5	94.4
GSTP1.C14, plasma	>1	5.55/0.645	22.2	37.5	44.1	27.8	62.5
GSTP1.C15, total	>1	8.27/5.92	63.2	0	40.5	26.3	55.6
GSTP1.C15, plasma	>1	5.54/1.03	5.6	18.8	32.4	0	68.8
GSTP1.C16, total	0.00000013	9.12/0.204	100	100	100	100	100
GSTP1.C16, plasma	>1	5.00/0.569	5.6	6.2	44.1	0	93.8
GSTP1.C17, total	0.00000013	8.11/0.136	100	100	100	100	100
GSTP1.C17, plasma	>1	4.96/0.413	5.6	6.2	52.9	16.7	93.8

The mean values are reported as mean in the PCa group/mean in the HD group. Sensitivity is the proportion of PCa patients correctly identified as such, and specificity is the proportion of HD patients correctly identified as such.

**Table 5:** Diagnostic value of individual cytosine or paired methylation cytosines status when PCa patients are compared with HD.

Option Number	Gene, Position, Status (C or T after Conversion)	*p*-Value × 1167	Means, % (PCa/HD)	Sensitivity for 100% Specificity, %	Specificity for 100% Sensitivity, %	Cut Off, % (Ratio)	CV Accuracy, %	CV Sensitivity, %	CV Specificity, %	CV AUC,% (DeLong’s CI)
1	GSTP1.C3	0.00000013	12.0/0.247	100	100	0.768 (4.53)	100	100	100	100
2	GSTP1.C11	0.00000013	12.6/0.155	100	100	0.586 (8.42)	100	100	100	100
3	GSTP1.T3.T5	0.00000013	87.5/99.7	100	100	98.8 (1.02)	100	100	100	100
4	GSTP1.T1.C6	0.00000013	4.69/0.232	100	100	0.607 (4.13)	100	100	100	100
5	GSTP1.C2.C3	0.00000013	7.51/0.0478	100	100	0.174 (1.99)	100	100	100	100
· · · · · · · · · · · · · · · · · · · · · · · · · · · · · · · · · · · · · · · · · · · · · · · · · · · · · · · · · · · · · · · · · · · · · · · · · · · · · · · · · · · · · · · · · · · · · · · · · · · · · · · · · · · · · · · · · · · · · · · · · · ·										
474	RNF219.C2.C4	0.00000013	0.301/0.00174	100	100	0.0226 (6.29)	100	100	100	100
475	RNF219.C3.C12	0.00000013	0.157/0.00132	100	100	0.0199 (3.30)	100	100	100	100
476	RNF219.C10.C14	0.00000013	0.483/0.00485	100	100	0.0574 (4.08)	100	100	100	100

The mean values are reported as mean in the PCa group/mean in the HD group. The cutoff value is an estimate for the threshold which may be good for separating the comparison groups. The corresponding ratio is reported in the case of complete separation, which is the ratio of minimum in the comparison group with higher values to the maximum in the comparison group with lower values. Sensitivity is the proportion of PCa patients correctly identified as such, and specificity is the proportion of HD patients correctly identified as such.

**Table 6:** Comparison of PCa patients with HD.

	Average in HD, %	Average in PCa, %	Cutoff, % (Ratio)
**Forward primer area**			
GSTP1.C5.C6	0.002	11.448	0.0909 (180.63)
**GSTP1.C4.C5.C6**	**0.002**	**11.361**	**0.0896 (175.54)**
GSTP1.C1.C2.C3	0.001	7.237	0.0299 (43.88)
GSTP1.C1.C2.C3.C4	0.001	7.221	0.0295 (42.85)
GSTP1.C1.C2.C3.C4.C5	0.001	7.211	0.0288 (40.65)
**TaqMan-probe area**			
GSTP1.C6.C7	0.002	11.608	0.104 (145.99)
GSTP1.C7.C8.C9.C10	0.001	6.724	0.0293 (50.48)
GSTP1.C8.C9.C10.C11.C12	0.001	6.718	0.0293 (50.48)
**GSTP1.C7.C8.C9.C10.C11**	**0.001**	**6.698**	**0.0289 (49.27)**
**GSTP1.C7.C8.C9.C10.C11.C12**	**0.001**	**6.683**	**0.0289 (49.27)**
GSTP1.C6.C7.C8	0.001	6.608	0.0282 (46.87)
GSTP1.C6.C7.C8.C9	0.001	6.592	0.0282 (46.87)
GSTP1.C6.C7.C8.C9.C10	0.001	6.579	0.0282 (46.87)
**Reverse primer area**			
GSTP1.C14.C15.C16	0.001	7.881	0.0289 (49.27)
**GSTP1.C15.C16.C17**	**0.001**	**7.878**	**0.0289 (49.27)**
**GSTP1.C14.C15.C16.C17**	**0.001**	**7.870**	**0.0289 (49.27)**

**Table 7:** The best combinations of cytosines for TaqMan PCR design.

	Average in HD, %	Average in PCa,%	Cutoff, % (Ratio)
GSTP1.C4.C5.C6.C7.C8.C9.C10.C11.C15.C16.C17	0.001	6.231	0.024 (35.5)
GSTP1.C4.C5.C6.C7.C8.C9.C10.C11.C12.C15.C16.C17	0.001	6.227	0.024 (35.5)
GSTP1.C4.C5.C6.C7.C8.C9.C10.C11.C14.C15.C16.C17	0.001	6.227	0.024 (35.5)
GSTP1.C4.C5.C6.C7.C8.C9.C10.C11.C12.C14.C15.C16.C17	0.001	6.223	0.024 (35.5)

**Table 8:** List of possible additional combinations of cytosines for the “creation” of PCR systems.

	Average in HD, %	Average in PCa, %
**Potential area of forward primer**		
GSTP1.C4.**T5**.C6	0.000630	0.0100
**Potential area of TaqMan-probe**		
GSTP1.C7.C8.**T9**.C10.C11	0	0.00281
GSTP1.C7.C8.C9.**T10**.C11	0	0.00657
GSTP1.C7.C8.**T9.T10**.C11	0	0.0118
GSTP1.C7.C8.**T9**.C10.C11.C12	0	0.00281
GSTP1.C7.C8.C9.**T10**.C11.C12	0	0.00594
GSTP1.C7.C8.**T9.T10**.C11.C12	0	0
**Potential missmatch molecules in tcfDNA**		
GSTP1.C4.**T5**.C6.C7.C8.**T9**.C10.C11.C15.C16.C17	0	0
GSTP1.C4.**T5**.C6.C7.C8.C9.**T10**.C11.C15.C16.C17	0	0
GSTP1.C4.**T5**.C6.C7.C8.**T9.T10**.C11.C15.C16.C17	0	0
GSTP1.C4.**T5**.C6.C7.C8.**T9**.C10.C11.C12.C15.C16.C17	0	0
GSTP1.C4.**T5**.C6.C7.C8.C9.**T10**.C11.C12.C15.C16.C17	0	0
GSTP1.C4.**T5**.C6.C7.C8.**T9.T10**.C11.C12.C15.C16.C17	0	0
GSTP1.C4.**T5**.C6.C7.C8.**T9**.C10.C11.C14.C15.C16.C17	0	0
GSTP1.C4.**T5**.C6.C7.C8.C9.**T10**.C11.C14.C15.C16.C17	0	0
GSTP1.C4.**T5**.C6.C7.C8.**T9.T10**.C11.C14.C15.C16.C17	0	0
GSTP1.C4.**T5**.C6.C7.C8.**T9**.C10.C11.C12.C14.C15.C16.C17	0	0
GSTP1.C4.**T5**.C6.C7.C8.C9.**T10**.C11.C12.C14.C15.C16.C17	0	0
GSTP1.C4.**T5**.C6.C7.C8.**T9.T10**.C11.C12.C14.C15.C16.C17	0	0
GSTP1.C4.C5.C6.C7.C8.**T9**.C10.C11.C15.C16.C17	0	0.00281
GSTP1.C4.C5.C6.C7.C8.C9.**T10**.C11.C15.C16.C17	0	0.00575
GSTP1.C4.C5.C6.C7.C8.**T9.T10**.C11.C15.C16.C17	0	0
GSTP1.C4.C5.C6.C7.C8.**T9**.C10.C11.C12.C15.C16.C17	0	0.00281
GSTP1.C4.C5.C6.C7.C8.C9.**T10**.C11.C12.C15.C16.C17	0	0.00575
GSTP1.C4.C5.C6.C7.C8.**T9.T10**.C11.C12.C15.C16.C17	0	0
GSTP1.C4.C5.C6.C7.C8.**T9**.C10.C11.C14.C15.C16.C17	0	0.00281
GSTP1.C4.C5.C6.C7.C8.C9.**T10**.C11.C14.C15.C16.C17	0	0.00575
GSTP1.C4.C5.C6.C7.C8.**T9.T10**.C11.C14.C15.C16.C17	0	0
GSTP1.C4.C5.C6.C7.C8.**T9**.C10.C11.C12.C14.C15.C16.C17	0	0.00281
GSTP1.C4.C5.C6.C7.C8.C9.**T10**.C11.C12.C14.C15.C16.C17	0	0.00575
GSTP1.C4.C5.C6.C7.C8.**T9.T10**.C11.C12.C14.C15.C16.C17	0	0

## Data Availability

Data cannot be shared publicly because of the Ethics committee of the Novosibirsk Regional Oncology Dispensary regulations. Deidentified data will be provided to any qualified investigator on reasonable request. Proposals will be reviewed and approved by the researchers, local regulatory authorities, and the ethics committee of the Novosibirsk Regional Oncology Dispensary. Once the proposal has been approved, data can be transferred through a secure online platform after the signing of a data access agreement and a confidentiality agreement.

## Supplementary Materials

The following supporting information can be downloaded at: https://www.mdpi.com/article/10.3390/cancers15020431/s1, Table S1: Diagnostic value of individual cytosine or paired methylation cytosines status when PCa patients are compared with HD. Figure S1. Schematic of the experimental part of the study.
