# Supplementary figures and images for: Locus-Specific Bisulfate NGS Sequencing of GSTP1, RNF219, and KIAA1539 Genes in the Total Pool of Cell-Free and Cell-Surface-Bound DNA in Prostate Cancer: A Novel Approach for Prostate Cancer Diagnostics
Source: Cancers (Basel). 2023 Jan 9;15(2):431. doi: 10.3390/cancers15020431 (PMC9856824; doi:10.3390/cancers15020431)

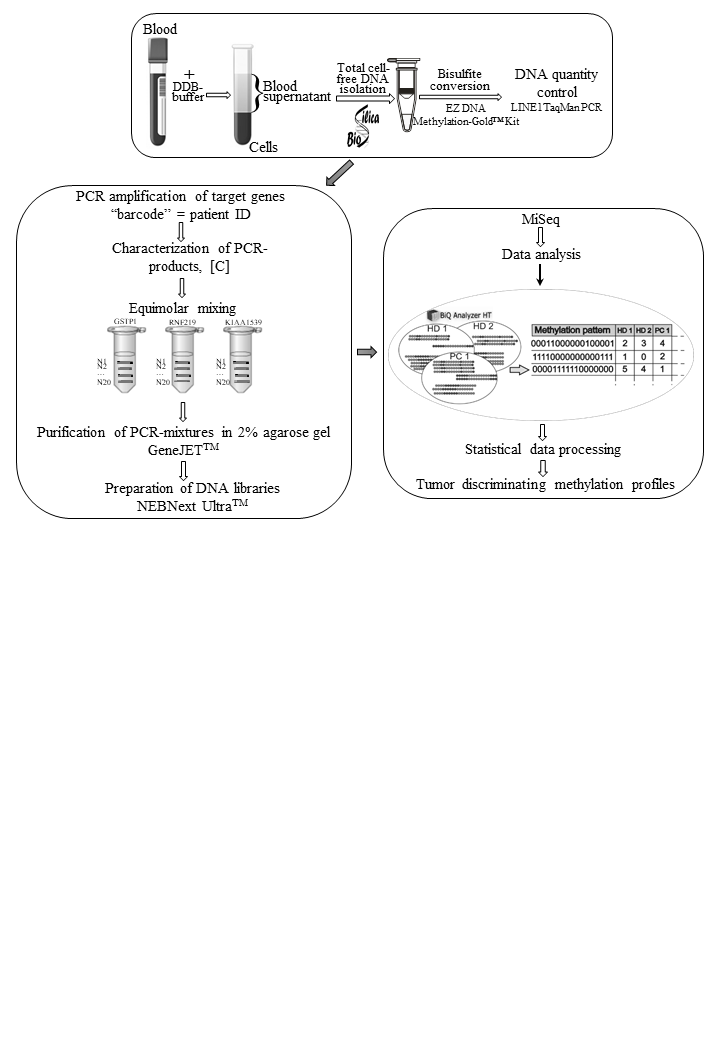

Supplement: Supplementary file 1 [file cancers-15-00431-s001.zip › Supplementary Figure S1.jpeg]
